# The oligonucleotides containing N7-regioisomer of guanosine: influence on thermodynamic properties and structure of RNA duplexes

**DOI:** 10.1261/rna.080106.124

**Published:** 2025-01

**Authors:** Aleksandra Jarmolowicz, Nivedita Dutta, Witold Andralojc, Joanna Sarzynska, Grzegorz Framski, Daniel Baranowski, Jerzy Boryski, Ansuman Lahiri, Zofia Gdaniec, Elzbieta Kierzek, Ryszard Kierzek

**Affiliations:** 1 Institute of Bioorganic Chemistry, Polish Academy of Sciences, 61-704 Poznan, Poland; 2 University of Calcutta, Kolkata-700009, West Bengal, India

**Keywords:** N7-guanosine, structure and thermodynamics, RNA duplexes, tautomers, NMR, molecular dynamics

## Abstract

During the chemical synthesis of the purine riboside, N7-regioisomer is kinetically formed, whereas N9-regioisomer is a thermodynamically formed product. We have studied the effect of substituting N9-regioisomer of guanosine with its N7-regioisomer (N7-guanosine, 7G) at a central position of several RNA duplexes. We found that this single substitution by 7G severely diminished their thermodynamic stabilities when 7G paired with C and U, but remarkably, led to a significant amount of stabilization in most of the duplexes when forming mismatches with G and A. The extent of stabilization was observed to be dependent on the sequence and orientation of neighboring base pairs of N7-guanosine. 1D and 2D NMR studies on the duplexes along with extensive molecular dynamics simulations revealed the conformational differences occurring due to the substitution of G by 7G, and it was observed that the thermodynamic results were largely explainable by considering the formation of stable noncanonical hydrogen bonding interactions, although other interactions such as stacking and electrostatic interactions could also play a role. These observations can have important applications in the design of RNA-based disease diagnostics and therapeutics.

## INTRODUCTION

**FIGURE 1.**
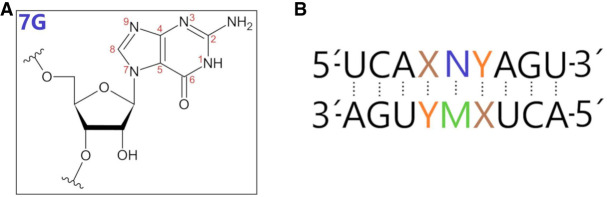
(*A*) The chemical structure of N7-guanosine with the numbering of atoms in the aromatic base. (*B*) General sequence of the 7G-duplexes. Here, *X* and *Y* form pairs G-C, C-G, A-U, and U-A. N represents 7G and M represents A, C, G, and U residues.

Acid-catalyzed ribosylation of heterocyclic bases leads to the formation of a ribosylated kinetic product, which, under suitable reaction conditions, undergoes isomerization to the final thermodynamic product. The formation of 7-ribofuranosylguanine (N7-guanosine, 7G) as a kinetic product during the chemical synthesis of guanosine has been known for years (Fig. 1; Watanabe et al. 1974). However, 7-regioisomers have been treated as an unwanted side-product, decreasing the desired material's yield. The knowledge about 7-regioisomer of guanosine (N7-guanosine) is minimal, and there have been just a few reports on its cytostatic or antiviral activities (Prekupec et al. 2005). More recently, we have discovered that 7-(β-d-ribofuranosyl)guanine demonstrates high and selective anticancer activity in vitro against glioma cell lines T98G (*Glioblastoma multiforme*) (Framski et al. 2016). This unexpected finding prompted us to look more closely at the structural properties of N7-guanosine at the monomer level and after incorporation into the RNA oligonucleotide chain.

Studies of the 7-regioisomer of guanosine could be important for several reasons, for example, its observed antivirus activity. In the cell, the nucleoside is enzymatically converted into triphosphate and incorporated into RNA. The studies presented here provide information on the possible pairing of N7-guanosine in the duplex context. Additionally, the unique base-pairing properties of 7G make that oligonucleotide a good candidate for inhibition biological studies targeting the pathogenic RNA at the single-nucleotide polymorphic (SNP) side. Moreover, nonstandard interactions of 7G within an oligonucleotide with A or G in the opposite strand provide additional information on the conformational flexibility of nucleotide residues within RNA.

There are a few reports on oligonucleotides containing 7-ribo- or 7-deoxyguanosine residues. A synthesis of oligonucleotides in which 2′-deoxyguanosine was replaced by its 7-regioisomer (7dG) was reported (Rao et al. 1994). This exchange did not enhance antiparallel triple helix formation in analyzed cases. Moreover, the oligonucleotides with the acyclic analog of 7G, which formed DNA triplexes, were also investigated (St. Clair et al. 1998). The authors concluded that N1H and NH2 of the modified unit could be involved in recognizing guanine residue in G-C base pairs. The ability of 7dG to bind G-C base pairs with a greater affinity and specificity than G and form a 7dG:G-C triplet has also been studied (Hunziker et al. 1995; Koshlap et al. 1997). The solution NMR structure of an intramolecular DNA triple helix containing 7dG (PDB ID: 1GN7) was also reported (Hunziker et al. 1995; Koshlap et al. 1997). More recently, the base-pairing properties of 7-deoxyguanine and its 9-deaza analog were compared in oligonucleotide duplexes and triplexes (Leonard et al. 2001). Moreover, data on incorporating N7-2′-deoxyadenosine and N7-2′-deoxy-3-deazaguanosine into DNA oligonucleotide were previously described (Seela and Leonard 1997; Seela and Zulauf 1999). Recently, it was reported that enzymatic glycosylation of xanthine results in the formation of the mixture of the N9 and N7 regioisomers of xanthosine. The N7-xanthosine performs many atypical physicochemical properties compared to N9-regioisomer (Westarp et al. 2024).

In the present work, we focus on thermodynamic and structural studies of RNA duplexes containing N7-guanosine residues at central positions. In those RNA duplexes, one strand contained exclusively natural nucleotide residues, whereas the second strand at the central position contained a single N7-guanosine (Fig. 1). We measured the thermodynamic stability of the duplexes containing 7G-C, 7G-U, 7G-A, and 7G-G pairs. Moreover, for each 7G-C, 7G-U, 7G-A, and 7G-G pair, we determined the influence of 5′- and 3′-adjacent base pairs (G-C, C-G, A-U, and U-A) on their thermodynamic stability. To explain the unexpected effect of 7G on the thermodynamic stability of RNA duplexes, we performed nuclear magnetic resonance (NMR) and molecular dynamic (MD) investigations for some RNA duplexes containing 7G-A and 7G-G adjacent to G-C and C-G pairs.

## RESULTS

Almost all natural nucleotides in RNA and DNA contain N-glycoside bonds between C1′ and N9 or N1 for purine and pyrimidine, respectively. The exception is pseudouridine and its methylated derivatives, which connect C1′ of sugar and C5 uridine (McCown et al. 2020). Herein, we studied RNA oligonucleotides containing the N7-regioisomer of guanosine.

### Thermodynamic stabilities of RNA duplexes containing N7-guanosine residue

Inversion of the guanosine base in N7-regioisomer analog could change base-pairing and thermodynamic stabilities of oligonucleotides containing this type of residue. To get more information on that issue, we need to know how the presence of the N7-guanosine within the RNA duplexes changes their thermodynamic stability and interactions when 7G pairs with A, C, G, or U in the opposite strand (Fig. 1; Table 1). We find that the presence of 7G-G and 7G-A mainly enhances the thermodynamic stability of RNA duplexes compared to analogous duplexes containing G-G and G-A mismatches, whereas 7G-C and 7G-U diminishes duplex stability.

**TABLE 1.** Thermodynamic parameters of RNA duplexes containing N7-guanosine and reference duplexes^a^

Duplexes (5′-3′)	The average of the curve fits				*T*_M_^−1^ versus log *C*_*T*_ plots					
	−Δ*H*° (kcal/mol)	−Δ*S*° (eu)	$-\Delta G_{37}^{\circ }$ (kcal/mol)	*T*_*M*_^b^ (°C)	−Δ*H*° (kcal/mol)	−Δ*S*° (eu)	$-\Delta G_{37}^{\circ }$ (kcal/mol)	*T*_*M*_^b^ (°C)	$-\Delta \Delta G_{37}^{\circ }$^d^ (kcal/mol)	Δ*T*_*M*_^b,d^ (°C)
5′ UCAGGCAGU 3′ AGUCCGUCA	80.0 ± 2.0	213.0 ± 5.9	13.93 ± 0.17	68.6	77.6 ± 0.7	206.0 ± 2.1	13.74 ± 0.06	68.7	0.00	0.00
5′ UCAG**7G**CAGU 3′ AGUC CGUCA	67.9 ± 3.4^c^	188.8 ± 10.3	9.33 ± 0.30	50.3	56.7 ± 4.3	153.7 ± 13.4	9.01 ± 0.13	51.2	4.73	17.5
5′ UCAGGCAGU 3′ AGUCUGUCA	83.5 ± 3.4	230.6 ± 10.4	12.03 ± 0.17	58.9	74.2 ± 1.4	202.0 ± 4.4	11.53 ± 0.08	59.4	0.00	0.0
5′ UCAG**7G**CAGU 3′ AGUC UGUCA	69.9 ± 2.6	198.5 ± 8.0	8.32 ± 0.11	45.2	69.7 ± 2.8	198.0 ± 8.8	8.32 ± 0.06	45.2	3.21	14.2
5′ UCAGGCAGU 3′ AGUCAGUCA	68.7 ± 8.3	194.2 ± 26.5	8.49 ± 0.15	46.1	69.3 ± 3.7	196.3 ± 11.8	8.45 ± 0.08	45.9	0.00	0.0
5′ UCAG**7G**CAGU 3′ AGUC AGUCA	73.0 ± 4.3	204.9 ± 13.2	9.49 ± 0.27	50.1	66.9 ± 4.7	185.6 ± 14.6	9.31 ± 0.17	50.4	−0.86	−4.5
5′ UCAGGCAGU 3′ AGUCGGUCA	77.3 ± 1.9	217.4 ± 5.8	9.92 ± 0.11	51.2	85.0 ± 2.5	240.9 ± 7.7	10.30 ± 0.12	51.4	0.00	0.0
5′ UCAG**7G**CAGU 3′ AGUC GGUCA	80.7 ± 9.1	222.6 ± 27.6	11.65 ± 0.51	58.0	73.9 ± 2.2	202.1 ± 6.6	11.25 ± 0.11	58.1	−0.95	−6.7
5′ UCACGGAGU 3′ AGUGCCUCA	86.9 ± 2.9	234.1 ± 8.6	14.34 ± 0.35	67.6	76.8 ± 6.7	204.2 ± 19.8	13.46 ± 0.60	67.7	0.00	0.0
5′ UCAC**7G**GAGU 3′ AGUG CCUCA	69.1 ± 7.4^c^	192.6 ± 22.5	9.35 ± 0.43	50.2	57.1 ± 2.9	155.7 ± 8.8	8.84 ± 0.11	50.1	4.62	17.6
5′ UCACGGAGU 3′ AGUGUCUCA	81.4 ± 6.3	226.4 ± 10.1	11.19 ± 0.40	55.8	78.2 ± 3.4	216.8 ± 10.5	11.01 ± 0.20	55.8	0.00	0.0
5′ UCAC**7G**GAGU 3′ AGUG UCUCA	73.8 ± 4.3	208.0 ± 13.2	9.31 ± 0.20	49.1	71.3 ± 4.7	200.3 ± 14.8	9.24 ± 0.20	49.2	1.77	6.7
5′ UCACGGAGU 3′ AGUGACUCA	62.5 ± 2.7	175.6 ± 8.5	8.01 ± 0.10	44.5	68.2 ± 7.7	193.9 ± 20.4	8.03 ± 0.20	44.0	0.00	0.0
5′ UCAC**7G**GAGU 3′ AGUGACUCA	74.8 ± 8.4	209.6 ± 26.4	9.77 ± 0.30	51.0	65.4 ± 1.9	180.4 ± 5.9	9.44 ± 0.10	51.4	−1.39	−6.5
5′ UCACGGAGU 3′ AGUGGCUCA	93.0 ± 11.6	268.2 ± 36.2	9.85 ± 0.41	48.5	84.8 ± 9.3	242.2 ± 29.3	9.72 ± 0.30	49.1	0.00	0.0
5′ UCAC**7G**GAGU 3′ AGUG GCUCA	88.8 ± 11.7^c^	247.1 ± 36.3	12.17 ± 0.46	58.0	74.4 ± 13.2	202.8 ± 40.1	11.48 ± 0.84	59.1	−1.76	−10.0
5′ UCAAGUAGU 3′ AGUUCAUCA	83.3 ± 4.4	234.8 ± 13.7	10.51 ± 0.22	52.6	75.7 ± 10.3	211.3 ± 31.8	10.51 ± 0.46	52.6	0.00	0.00
5′ UCAA**7G**UAGU 3′ AGUU CAUCA	53.6 ± 9.6	154.9 ± 31.6	5.50 ± 0.19	31.2	60.2 ± 6.1	176.7 ± 20.3	5.40 ± 0.19	31.3	5.11	21.3
5′ UCAAGUAGU 3′ AGUUUAUCA	68.2 ± 4.1	197.1 ± 12.9	7.10 ± 0.10	39.6	72.7 ± 3.4	211.6 ± 10.9	7.08 ± 0.10	39.4	0.00	0.0
5′ UCAA**7G**UAGU 3′ AGUU UAUCA	69.3 ± 16.8	204.4 ± 55.1	5.90 ± 0.30	34.2	64.1 ± 12.2	187.5 ± 39.7	5.93 ± 0.44	34.1	1.16	5.4
5′ UCAAGUAGU 3′ AGUUAAUCA	43.9 ± 1.5	127.0 ± 4.5	4.50 ± 0.10	23.4	43.8 ± 6.6	126.6 ± 22.4	4.52 ± 0.50	23.4	0.00	0.0
5′ UCAA**7G**UAGU 3′ AGUU AAUCA	52.2 ± 7.2	152.7 ± 23.5	4.80 ± 0.20	27.2	50.4 ± 7.5	146.8 ± 24.8	4.88 ± 0.40	27.2	−0.36	−3.8
5′ UCAAGUAGU 3′ AGUUGAUCA	40.2 ± 7.4	108.7 ± 23.9	6.45 ± 0.16	36.4	46.0 ± 3.4	127.5 ± 11.2	6.43 ± 0.11	36.3	0.00	0.0
5′ UCAA**7G**UAGU 3′ AGUU GAUCA	65.0 ± 4.7	184.3 ± 14.5	7.87 ± 0.28	43.5	57.3 ± 9.9	159.8 ± 31.5	7.77 ± 0.38	43.9	−1.34	−7.6
5′ UCAUGAAGU 3′ AGUACUUCA	85.1 ± 3.7	240.3 ± 11.3	10.56 ± 0.22	52.4	76.2 ± 3.1	212.7 ± 9.5	10.24 ± 0.11	52.9	0.00	0.0
5′ UCAU**7G**AAGU 3′ AGUA CUUCA	46.8 ± 6.2	135.2 ± 20.6	4.85 ± 0.24	26.2	46.0 ± 3.2	132.7 ± 10.9	4.86 ± 0.17	26.1	5.38	26.8
5′ UCAUGAAGU 3′ AGUAUUUCA	52.8 ± 9.2	148.2 ± 30.2	6.48 ± 0.29	38.8	49.5 ± 7.9	137.2 ± 25.9	6.95 ± 0.44	39.6	0.00	0.0
5′ UCAU**7G**AAGU 3′ AGUA UUUCA	57.1 ± 3.6^c^	170.5 ± 12.5	4.23 ± 0.36	25.0	66.9 ± 19.4	204.0 ± 65.3	3.68 ± 0.30	24.3	3.27	13.8
5′ UCAUGAAGU 3′ AGUAAUUCA	60.2 ± 8.4	170.1 ± 25.9	7.35 ± 0.24	41.3	58.4 ± 8.5	132.1 ± 28.7	7.48 ± 0.80	43.2	0.00	0.0
5′ UCAU**7G**AAGU 3′ AGUA AUUCA	48.6 ± 2.0	143.9 ± 6.7	3.97 ± 0.13	21.5	53.0 ± 5.4	158.9 ± 18.5	3.72 ± 0.35	21.4	3.76	21.8
5′ UCAUGAAGU 3′ AGUAGUUCA	62.1 ± 8.1	175.9 ± 26.1	7.58 ± 0.29	42.3	54.7 ± 4.6	152.5 ± 14.8	7.44 ± 0.13	42.2	0.00	0.0
5′ UCAU**7G**AAGU 3′ AGUA GUUCA	49.3 ± 6.4	135.9 ± 20.9	7.17 ± 0.18	41.1	54.1 ± 2.5	151.3 ± 7.9	7.21 ± 0.04	40.9	0.23	1.3

^a^Solutions 1 M sodium chloride, 20 mM sodium cacodylate, 0.5 mM Na_2_EDTA, pH 7.

^b^Calculated for 10^−4^ M oligomer concentration.

^c^Duplexes not performing two-state melting.

^d^The free energies and the melting temperatures of 7G duplexes were compared to respective duplex containing guanosine.

For the thermodynamic studies, we synthesized several oligonucleotides that formed RNA duplexes: 5′UCAX**N**YAGU/3′AGUY**M**XUCA, where XY means pairs G-C, C-G, A-U, U-A, whereas N corresponds to 7G and M indicates A, C, G, or U (Fig. 1; Table 1). In consequence, 7G formed base pair 7G-C and mismatches with 7G-U, 7G-A, and 7G-G. For many single and tandem RNA mismatches, the sequences and orientations of 5′- and 3′-adjacent base pairs have been reported to influence the thermodynamic stability of RNA duplexes (Wu et al. 1995; Kierzek et al. 1999). This observation is usually related to the different stacking abilities of the adjacent nucleotides and the distribution of electrostatic potential in the functional groups of neighboring nucleobases. In the present work, we performed similar studies in the case of 7G RNA duplexes. In each case, the contribution of 7G to thermodynamic stabilities was compared to RNA duplexes with G (N9-regioisomer).

Thermodynamics investigations indicated that the presence of 7G-C and 7G-U pairs strongly destabilizes RNA duplexes, whereas pairing of 7G with purine riboside (forming 7G-A and 7G-G pairs) in most cases stabilizes mismatched RNA duplexes (Tables 1 and 2). Analysis of Table 2 revealed that the thermodynamic effect of replacing G with 7G largely depends on the kind of base pair they formed and on the sequence and orientation of adjacent base pairs. When 7G pairs with C or U (forming 7G-C and 7G-U pairs), it results in a diminished thermodynamic stability (ΔΔ$G_{37}^{\circ }$) of RNA duplexes in the range of 1.15–5.38 kcal/mol, regardless of the nature of the adjacent base pairs. At the same time, pairing of 7G with A or G (formation of 7G-A and 7G-G mismatches) results in enhancement of the thermodynamic stability of 7G RNA duplexes in the range of 0.36–1.76 kcal/mol when adjacent base pairs are 5′G-C/3′C-G, 5′C-G/3′G-C, and 5′A-U/3′U-A. However, 7G-A and 7G-G mismatches adjacent to 5′U-A/3′A-U resulted in a diminished thermodynamic stability of 7G RNA duplexes by 3.76 and 0.23 kcal/mol, respectively. Reduction of the thermodynamic stability of RNA duplexes carrying 7G-C or 7G-A by 1.15–5.38 kcal/mol suggests significant changes in hydrogen bonding and stacking interactions of both 7G mismatches. Considering that a single hydrogen bond in RNA contributes 1–1.5 kcal/mol to free energy (Turner et al. 1987), the drastic reduction in the stability of the duplex due to the replacement of G-C with 7G-C by 4.73 kcal/mol suggests a loss of all three hydrogen bonds within the 7G-C pair in the case of 7G-C being sandwiched between 5′GC/3′CG pairs (Table 2). Similarly, in the case of a duplex with 7G-U pair sandwiched between 5′GC/3′CG pairs, the destabilization was by 3.21 kcal/mol (Table 2), which was approximately equivalent to the loss of two hydrogen bonds (Turner et al. 1987). On the other hand, for RNA duplexes containing 7G-A and 7G-G mismatches nested between 5′GC/3′CG pairs, the thermodynamic stability was enhanced by 0.86 and 0.95 kcal/mol (ΔΔ$G_{37}^{\circ }$), respectively. It suggests the formation of an additional hydrogen bond compared to duplexes containing G-A and G-G. Previous studies indicate the involvement of two hydrogen bonds in G-A and G-G mismatches (SantaLucia et al. 1990, 1991; Kierzek et al. 1999).

**TABLE 2.** Comparison of the influence of 5′- and 3′-adjacent base pairs on the thermodynamic stability of RNA duplexes containing 7G

Interacting base pairs	5′- and 3′-adjacent base pairs											
	5′GC/3′CG			5′CG/3′GC			5′AU/3′UA			5′UA/3′AU		
	$-\Delta G_{37}^{\circ }$^a^ (kcal/mol)	$-\Delta G_{37}^{\circ }$^b^ (kcal/mol)	$\Delta \Delta G_{37}^{\circ }$^c^ (kcal/mol)	$-\Delta G_{37}^{\circ }$^a^ (kcal/mol)	$-\Delta G_{37}^{\circ }$^b^ (kcal/mol)	$\Delta \Delta G_{37}^{\circ }$^c^ (kcal/mol)	$-\Delta G_{37}^{\circ }$^a^ (kcal/mol)	$-\Delta G_{37}^{\circ }$^b^ (kcal/mol)	$\Delta \Delta G_{37}^{\circ }$^c^ (kcal/mol)	$-\Delta G_{37}^{\circ }$^a^ (kcal/mol)	$-\Delta G_{37}^{\circ }$^b^ (kcal/mol)	$\Delta \Delta G_{37}^{\circ }$^c^ (kcal/mol)
7G-C	13.74	9.01	4.73	13.46	8.84	4.62	10.51	5.40	5.11	10.24	4.86	5.38
7G-U	11.53	8.32	3.21	11.01	9.24	1.77	7.08	5.93	1.15	6.95	3.68	3.27
7G-A	8.45	9.31	−0.86	8.03	9.44	−1.39	4.52	4.88	−0.36	7.48	3.72	3.76
7G-G	10.30	11.25	−0.95	9.72	11.48	−1.76	6.43	7.77	−1.34	7.44	7.21	0.23

^a^Free energy of duplexes containing G.

^b^Free energy of duplexes containing 7G.

^c^Free energies ($\Delta G_{37}^{\circ }$) calculated with MeltWin 3.5, using 1/*T*_*M*_ versus log *C*_*T*_ plots, were used to compare the thermodynamic stability of duplexes.

### NMR conformational analysis of 7G-A and 7G-G containing duplexes

**FIGURE 2.**
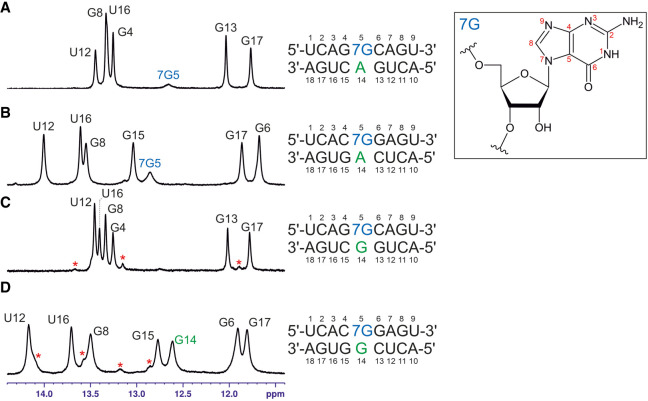
The imino proton regions of the ^1^H NMR spectra of the studied duplexes. (*A*) The 7G-A mismatch in 5′-G7GC/5′-GAC sequential context, (*B*) the 7G-A mismatch in 5′-C7GG/5′-CAG sequential context, (*C*) the 7G-G mismatch in 5′-G7GC/5′-GGC sequential context, (*D*) the 7G-G mismatch in 5′-C7GG/5′-CGG sequential context. All spectra were recorded at 15°C. For both **7G**-G containing duplexes, a second, minor spectral form was present; its imino resonances are marked in red stars.

To provide some atomic level justification for the stabilizing effect of 7G in the contexts studied using thermodynamics experiments, we studied two RNA duplexes each containing a 7G-A mismatch (Fig. 2A,B) and another two with 7G-G mismatches using NMR (Fig. 2C,D). In all the studied systems, the central mismatches were surrounded by G-C base pairs in two configurations.

**FIGURE 3.**
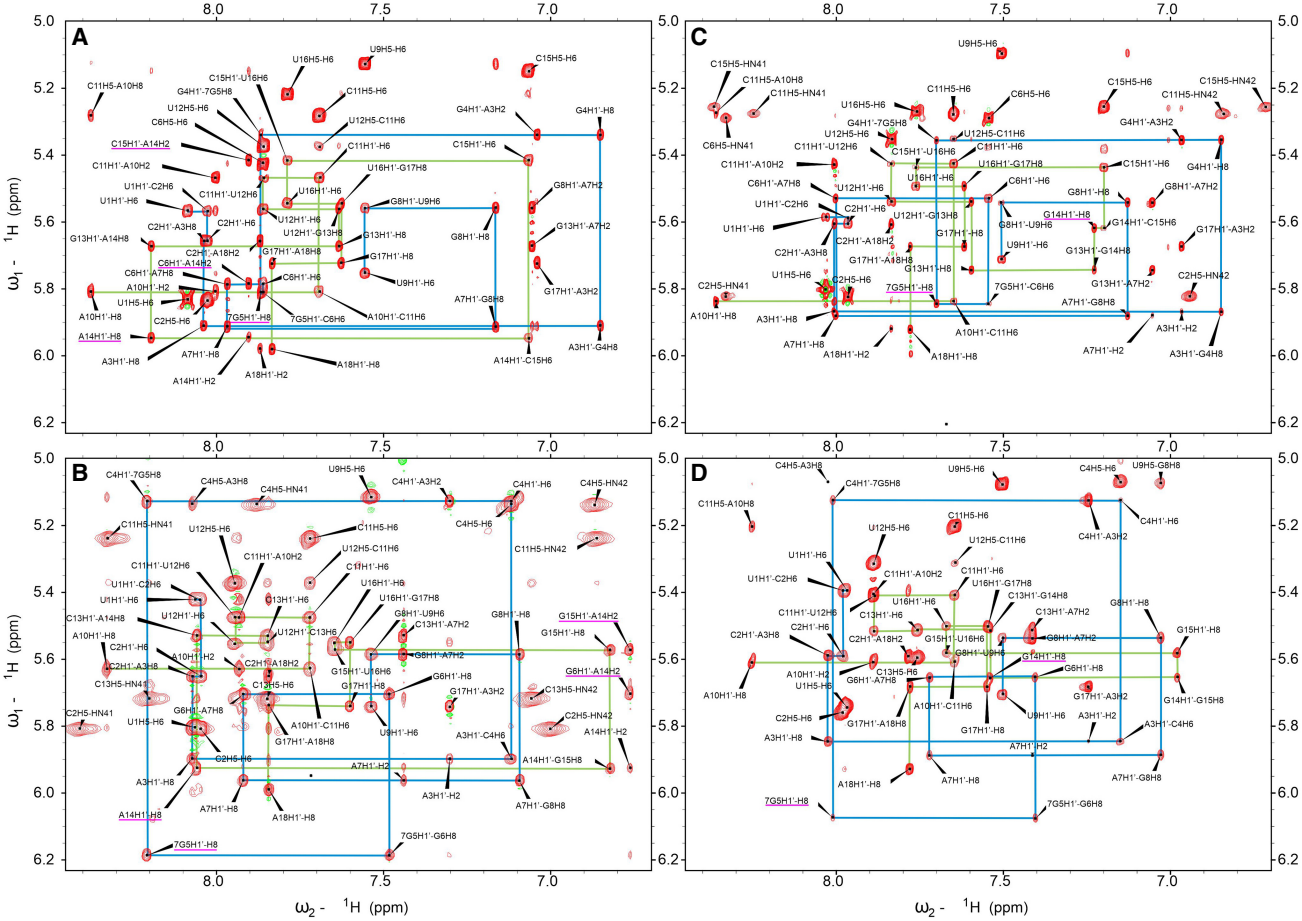
The aromatic-to-anomeric regions of the 2D ^1^H-^1^H NOESY spectra recorded for duplexes containing: (*A*) the **7G**-A mismatch in the 5′G**7G**C/3′C**A**G context, (*B*) the **7G**-A mismatch in the 5′C**7G**G/3′G**A**C context, (*C*) the **7G**-G mismatch in 5′G**7G**C/3′C**G**G context, and (*D*) the **7G**-G mismatch (in 5′C**7G**G/3′G**G**C context). Significant NOEs discussed in the text are marked in magenta. Sequential connectivity paths for the two strands are displayed in green and blue lines. For complete sequences and residue numbering, see Figure 1.

For both duplexes bearing a 7G-A mismatch, standard H1′-H8 NOESY (nuclear Overhauser effect spectroscopy) connectivity patterns (*NOESY-walks*) are observed (Fig. 3A,B). The intensities of H1′-H8 NOE cross peaks for the residues forming the mismatches (7G5 and A14) are close to the averages observed throughout the given duplex, which signifies that both 7G-A mismatches assume the *anti-anti* glycosidic state. In the imino proton region of the ^1^H NMR spectrum, six sharp and one very broad resonance can be observed for duplex A (Fig. 2A). A similar pattern is also observed for duplex B, yet the broadening of the seventh resonance is less severe. In both cases, the six sharp peaks correspond to imino protons belonging to standard Watson–Crick pairs (assignments given in Fig. 2A,B; terminal A-U pairs not observed due to solvent exchange), while the broader resonance can be assigned to 7G of the central mismatch based on its strong NOE cross peaks to A14-H2 observed in both duplexes. The chemical shift of the broadened resonance suggests its involvement in hydrogen bonding (free guanosine imino protons are found around 10–11 ppm), yet its linewidth indicates that it still remains rather labile. In contrast, another exchangeable proton resonance observed at 6.60 ppm (15°C duplex A; 6.52 ppm for duplex B) is very well protected from the solvent exchange, as it remains relatively sharp even at 45°C (Supplemental Fig. S1) when duplex melting is already visible in the NMR spectra. This resonance can be assigned to the amino group of A14 based on its attached nitrogen shift of 82.6 ppm (duplex A; 81.5 ppm for duplex B), characteristic for adenosine amino groups (Supplemental Fig. S2), as well as NOE contacts to exchangeable protons of both G-C base pairs flanking the central mismatch. Interestingly, its strongest NOE contact is yet another exchangeable proton, resonating at 5.85 ppm (duplex A; 5.71 ppm for duplex B). This group also produces NOE contacts to the C6-G13 flanking base pair and has the attached nitrogen shift equal to 70.0 ppm (Supplemental Fig. S2). Such a ^15^N shift is most compatible with a guanosine amino group and, as similar groups of Watson–Crick paired guanosines are rarely observable (Schnieders et al. 2019), it likely belongs to the 7G residue. Overall, among the exchangeable proton resonances, the amino group of A14 appears to be the most protected from solvent exchange—likely through stable hydrogen bonding—and is close to the 7G5 amino group. In contrast, the imino proton of 7G5, although rather labile, is also likely hydrogen bonded and lies near A14-H2. Some additional information about the geometry of the mismatch is provided by NOE contacts between the A14-H2 resonance and the C6-H1′, C15-H1′, and G13-H1 atoms (duplex A; G6-H1′, G15-H1′ atoms for duplex B) belonging to neighboring base pairs. All these NOEs have intensities slightly above those of H1′-H8 cross peaks and thus most likely correspond to distances <4.5 Å. For duplex B, weaker peaks to G6-H1, G15-H1, and C13-NH_2_ are also observed. Such a set of observations points toward a stable base-pairing arrangement between 7G5 and A14, yet is difficult to satisfy by any single 7G-A geometry (see discussion below). Moreover, the same pattern of observations repeats itself nearly perfectly in the two duplexes. One can thus conclude that the conformation of the 7G-A mismatch is conserved between the two studied sequential contexts.

Two more duplexes were analyzed to study the 7G-G pairing (Fig. 2C,D). The H1′-H8 NOE connectivities (Fig. 3) indicate that in both contexts, the 7G-G mismatches exist in the *anti-anti* glycosidic conformation. On the other hand, the exchangeable proton patterns appear to be context-specific. Only six imino protons are observed for the 5′G**7G**C/3′C**G**G duplex, corresponding to the six standard Watson–Crick pairs (Fig. 2C). Also, only the resonances of Watson–Crick paired cytosines are observed in the amino region. In contrast, for the 5′C**7G**G/3′G**G**C duplex, seven sharp imino resonances are present (Fig. 1D). Six among them can again be assigned to Watson–Crick pairs, yet the last has to belong to the central 7G-G mismatch, as it produces NOE contacts to G6 and G15 imino protons. Unambiguously assigning this resonance of either 7G5 or G14 would be impossible using standard methods, and thus, we used site-specific isotopic enrichment (see Supplemental Materials and Methods and Fig. S3) to conclude that this resonance belongs to G14. This resonance indicates that the G14 imino proton is involved in stable hydrogen bonding. This information is not enough, though, to confidently propose the 7G-G interaction geometry, for which reason the system was further studied using MD methods.

### Theoretical comparison of the stabilities of the N7-methylguanine and N9-methylguanine tautomers

**FIGURE 4.**
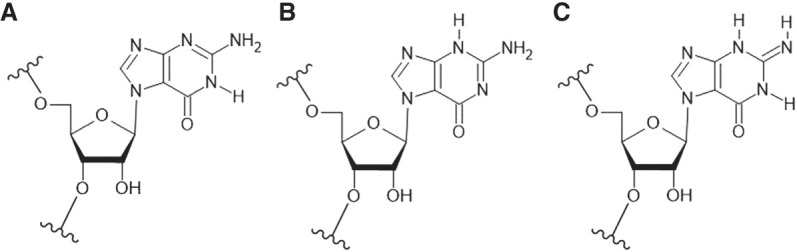
Chemical structures of the tautomers considered for MD simulations of 7G-duplexes, where (*A*) is 7G-N1H, (*B*) is 7G-N3H, and (*C*) is 7G-N1H,N2-imino tautomer.

Quantum chemical calculations performed for N7-methylguanine (m^7^Gua) using DFT methods have indicated that the keto-N1H-tautomer (Fig. 4) is the most stable tautomer both in the gas phase and in aqueous solution, while the enol-*cis* tautomer was found to be the least stable in both phases (Supplemental Figs. S4 and S5). The second-most stable tautomers were N2-imino forms in the gas phase and keto-N3H form in the solvent phase (that was predicted to be ∼5 kcal/mol less stable than the canonical keto-N1H tautomer). The trends in the stabilities of the m^7^Gua tautomers differed from that of the reference m^9^Gua tautomers. However, the N1H-tautomer of m^9^Gua was also observed to be the most stable in both gas and solvent phases, whereas the second most stable tautomer was the *trans*-enol form. Based on the observed stabilities of the m^7^Gua tautomers (representing N7G tautomeric forms), the keto-N1H, keto-N3H, and N1H,N3H,N2-imino-1 tautomers were chosen for studying the base-pairing geometries for N7G-A, N7G-G base pairs (the tautomers are referred to as 7G(N1H), 7G(N3H), 7G(N1H,N3H,N2-imino), respectively, for the nucleotide forms later in the text). The consideration of tautomers was aimed to explain NMR observations regarding observable exchangeable protons of the 7G base.

### Molecular dynamics analyses of 7G-A and 7G-G containing duplexes

**FIGURE 5.**
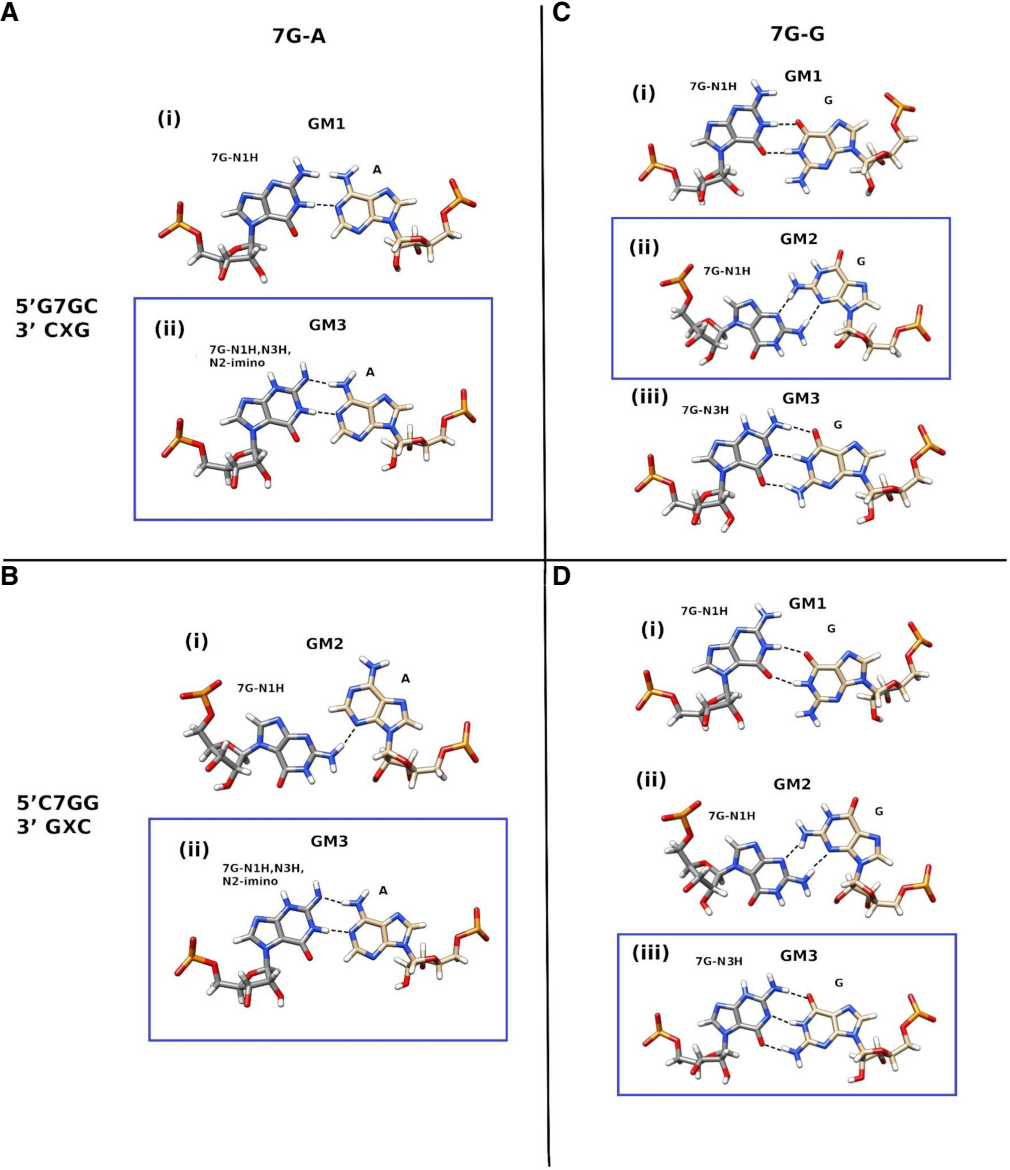
Observed geometries of 7G-A (*left: A,B*) and 7G-G (*right: C,D*) for the 5′G7GC/3′CXG (*up*: *A,C*) and 5′C7GG/3′GXC (*down*: *B,D*) contexts (X indicates A or G). The proposed geometries for the 7G-A and 7G-G pairs corresponding to the different contexts are shown within blue rectangles.

In MD simulation, for the 5′G**7G**C/3′C**A**G context (Fig. 2, duplex A), the 7G(N1H)-A base pair was observed to contain only a single hydrogen bond (7G)N1-HN1—N1(A) [GM1, where GM indicates geometry, Fig. 5Ai; Supplemental Fig. S6A(1a)]. Since the NMR data suggest an involvement of the amino group in hydrogen bonding, examination of the hypothesis that the N2-imino form of 7G [7G(N1H,N3H,N2-imino)] can better explain NMR observables (Fig. 5Aii) was performed.

The 7G(N1H,N3H,N2-imino) tautomer formed two hydrogen bonds to A14, i.e., (7G)N1-HN1—N1(A) and (A)N6-H62—N2(7G), whereas the geometry (GM3) parameters are similar in both scenarios and consistent with the NMR observances (Fig. 5Aii). In MD simulations, the imino proton of 7G is at average distances of 2.5 Å and 2.8 Å to A14-H2 in 7G(N1H) and 7G(N1H,N3H,N2-imino) form, respectively. The other short distances from A14-H2 to the C6-H1′, C15-H1′, and G13-H1 (Supplemental Table S1A) also explain the measured NOE pattern. Comparison of the stacking energies revealed that with the 7G(N1H,N3H,N2-imino) tautomer, the stacking interactions were more stable by ∼4 kcal/mol for the G-C/7G-A base pair step and ∼1.6 kcal/mol less stable for the 7G-A/C-G step than what was observed with the 7G(N1H) tautomer (Supplemental Fig. S6A; Supplemental Table S2A). The H-bond occupancy for N1H—N1 was observed to be 61%–62% in both cases (Supplemental Table S3A). The A14-N2H—N2-7G H-bond was maintained with occupancy of 49%.

A different geometry (GM2) was observed for the 7G-A base pair with 7G(N1H) for the 5′C**7G**G/3′G**A**C context (Fig. 2, duplex B), which was observed to contain one [(7G)N2-H21—N3(A)] hydrogen bond (Fig. 5Bi) in the 1 µsec exploratory simulation of the duplex. For a better estimation of the stabilities of these geometries in the two contexts, we simulated the duplex for another set of 1.1 µsec by restraining the 7G-A geometry to GM2 for duplex 5′G**7G**C/3′C**A**G and to GM1 for duplex-5′C7**G**G/3′G**A**C for the first 100 nsec and without such restraints in the next 1 µsec simulation. Our observations indicated that the geometry corresponding to one sequence context was not stable in the other context after removing the distance restraints for the 7G5-A14 base pair. However, with the 7G(N1H,N3H,N2-imino) tautomer, the same stable geometry (GM3) with similar hydrogen-bond frequencies was observed for both sequence contexts (Fig. 5Bi-ii; Supplemental Tables S2A and S3A). The molecular mechanics stacking interaction energy was more favorable for 7G-A (GM3) in the 5′G**7G**C/3′C**A**G sequence context (Supplemental Table S2A) than for 5′C**7G**G/3′G**G**C.

Water-mediated interactions also stabilized the 7G-A base-pairing geometries (Supplemental Fig. S7A). The hydration patterns differed for 7G-A GM1 for 5′G**7G**C/3′C**A**G and GM2 for duplex 5′C**7G**G/3′G**A**C. However, similar arrangements of bridging water molecules were observed for GM3, i.e., with the 7G(N1H,N3H,N2-imino) tautomer for each sequence context.

From the analyses of the NOE distances from the simulated ensembles, we observed that the GM3 geometry formed by the 7G(N1H,N3H,N2-imino) tautomer for each of the duplex contexts was in general better agreement than what was observed with the 7G(N1H) tautomer.

Our molecular mechanics/3D reference interaction site model (MM/3D-RISM) calculations of hybridization energy indicated that the duplex with the 7G(N1H,N3H,N2-imino) tautomer was more stable than the duplex with 7G(N1H) in both sequence contexts (Supplemental Table S2A).

Two different geometries were also observed for the 7G-G base pairs with 7G( N1H) for the 5′G**7G**C/3′C**G**G (Fig. 2, duplex C) and 5′C**7G**G/3′G**G**C (Fig. 2, duplex D) contexts, in the 1 µsec exploratory simulations, each containing two hydrogen bonds (Fig. 5Cii, Di). The first geometry (GM1) corresponding to C**7G**G context, contained the (7G)N1-H1—O6(G) and (G)N1-H1—O6(7G) H-bonds (Fig. 5Di) and the second geometry (GM2) observed for 5′G**7G**C/3′C**G**G, contained the (G)N2-H21—N3(7G) and (7G)N2-H22—N3(G) H-bonds (Fig. 5Cii). Hence, we performed another set of 1.1 µsec simulations of the duplexes by restraining the 7G-G geometry to GM2 for duplex 5′C**7G**G/3′G**G**C (Fig. 5Dii) and to GM1 for duplex 5′G**7G**C/3′C**G**G (Fig. 5Ci) for the first 100 nsec and without such restraints in the next 1000 nsec simulation (Supplemental Table S1B). The respective geometries (Fig. 5Ci, Dii) were stable during the simulation, even after removing the restraints.

For the 5′G**7G**C/3′C**G**G duplex, the GM2 geometry with two hydrogen bonds between the amino groups and N3 atoms and the exchangeable imino protons of 7G5 and G14 not involved in hydrogen bonding are in line with NMR results. Also, the stacking interactions were overall more favorable for the 7G-G in GM2 geometry (Supplemental Table S2B; Supplemental Fig. S6B).

For the 5′C**7G**G/3′G**G**C duplex, the GM1 geometry agrees with the NMR observation that indicates the involvement of the imino proton of G14 in hydrogen bonding. However, in this geometry, the imino proton of N7G is hydrogen bonding to O6 of A14, whereas this imino proton is not observed in NMR. We propose the N3H form of 7G to reconcile NMR and MD observables. In MD simulations, the 7G(N3H) tautomer forms three stable hydrogen bonds to G14 (GM3), similar to the canonical Watson–Crick C-G base pair (Fig. 5Diii). Tautomer 7G-N3H also formed a similar geometry with G in the 5′G**7G**C/3′C**G**G duplex (Fig. 5Ciii). Moreover, in GM1 and GM3 geometries, the distance between the imino proton of G14 and the imino proton of G6 and the distances between the imino proton of G14 and the H8 protons of G6 and G15 residues are within 6 Å, which explains the NOE contact between these atoms as seen in NMR (Supplemental Table S1B).

Additional water-mediated interactions were also observed to stabilize the 7G-G base-pairing geometries (Supplemental Fig. S7B). The hydration patterns differed for the three 7G-G geometries (GM1-3), but the arrangements were similar for each sequence context.

Although our results indicated the possibility of the formation of both these geometries for the **7G-G** base pair, GM1 was found to form more stable hydrogen bonding/base-pairing interactions than GM2 (by ∼8 kcal/mol) for each context (Supplemental Table S2B). The 7G-N3H tautomer formed the most stable base pair with G with three hydrogen bonds [(G)N2-H21—O6(7G), (G)N1-H1—N1(7G), and (7G)N2-H21—O6(G)] for each context (Supplemental Table S3B). The presence of (G)N1-H1—N1(7G) hydrogen bond agrees with the NMR observation.

Calculated stacking interactions (considering total stacking energies contributions from base pair step 1 and base pair step 2) were more favorable for 7G(N1H) (GM2 geometry) than for 7G(N3H) (GM3 geometry) by 2.2 kcal/mol and 3.5 kcal/mol for the G7GC and C7GG sequence context, respectively (Supplemental Table S2B). In the G**7G**C context, the least stable stacking interactions displayed 7G(N1H) in GM1 geometry, whereas in the G**7G**C context, they were more similar to that for GM3 geometry.

Our MM/3D-RISM hybridization energy calculations do not conclusively resolve which 7G-G pair geometry for 7G(N1H) (GM2 or GM1) in a 5′G**7G**C/3′C**G**G duplex leads to greater duplex stability. However, the computational predictions of hybridization energies for RNA duplexes with central 7G paired with purines display that for the 5′C**7G**G/3′G**G**C duplex, the 7G(N3H) tautomer (GM3 geometry) stabilizes this duplex the most (Supplemental Table S2B). This agrees with experimental results showing the most considerable stabilization of RNA duplex when 7G is paired with G in the 5′C**7G**G/3′G**G**C context (ΔΔ$G_{37}^{\circ }$ = −1.76 kcal/mol, Table 1). The MM/3D-RISM calculations revealed the overall stabilization of the duplexes over similar duplexes containing the **G-G** (*anti-syn/syn-anti*) pair for the C**G**G and G**G**C contexts (Supplemental Table S2B).

## DISCUSSION

The results presented indicate that N7-regioisomer of guanosine contributes differently to the thermodynamic stability of RNA duplexes than the natural N9-regioisomer.

The presented results can be discussed in various ways. The first one concerns the issue: how does the inversion of the guanosine to N7-guanosine change the hydrogen bonding and stacking interaction with pyrimidine and purine nucleotides in opposite strands of RNA duplexes? The second category concerns the possibility of application of those unusual thermodynamic properties of N7-guanosine in oligonucleotide-based therapy.

Thermodynamic results indicate that placing the N7-guanosine opposite to C or U results in strong RNA duplex destabilization, which could suggest a lack of stable hydrogen bonding and perhaps some steric destabilization effect. The nature of the 7G destabilization effect is dependent on 5′- and 3′-adjacent base pairs. Thermodynamic results concerning RNA duplexes containing 7G-A or 7G-G mismatches are very interesting. In this case, duplexes with 7G-A and 7G-G adjacent to 5′G-C/3′C-G, 5′C-G/3′G-C, and 5′A-U/3′U-A are more stable than duplexes carrying **G-A** and **G-G** mismatches. The situation is the opposite for duplexes with 7G-A surrounded by 5′U-A/3′A-U, and those with G-A are more stable. On the other hand, the thermodynamic stabilities of RNA duplexes carrying 7G-G and G-G mismatches adjacent to 5′U-A/3′A-U are very similar (Table 1).

To get insight into the origin of the stabilities of RNA duplexes with 7G-A and 7G-G surrounded by 5′G-C/3′C-G, 5′C-G/3′G-C, various types of NMR analyses and MD calculations involving the duplexes: 5′G**7G**C/3′C**A**G, 5′C**7G**G/3′G**A**C, 5′G**7G**C/3′C**G**G, and 5′C**7G**G/3′G**G**C were performed. We were particularly interested in the determination of the hydrogen bond interactions within **7G-A** and **7G-G** and stacking interactions with those 7G involved pairs with 5′- and 3′-adjacent 5′G-C/3′C-G and 5′C-G/3′G-C (Table 1; Figs. 2 and 3). Results presented herein concern the thermodynamic stabilities of RNA duplexes containing single N7-regioisomers of guanosine at the central position. A strong diminishing of the thermodynamic stability of those RNA duplexes when 7G interacts with any nucleotide residue in the opposite strand could be expected. On the other hand, enhancement of the thermodynamic stability of RNA duplex when 7G pairs to A and G was challenging to predict. This suggested the formation of some very noncanonical interactions within **7G-A** and **7G-G** pairs. NMR and MD methods were applied to solve the nature of those interactions. In most cases, schemes of hydrogen interactions postulated by both these approaches fit together when we considered tautomeric forms of 7G (Singh et al. 2015). Overall, from our observations based on NMR and MD simulations, in general, it can be inferred that for the 7G-A base pair, the 7G(N1H,N3H,N2-imino) tautomeric form explains experimental observables for the studied duplex contexts (Fig. 5A,B). On the other hand, for the 7G-G base pair, the possibility of two different 7G-tautomers, i.e., the 7G(N1H) tautomer for the 5′G**7G**C/3′C**G**G context (Fig. 5C) and the 7G-N3H tautomer for the 5′C**7G**G/3′G**G**C (Fig. 5D) context could be postulated.

The fact that 7G within an oligonucleotide makes a stronger pair with A and G than guanosine suggests the possible application of modified oligonucleotides. For example, single-nucleotide polymorphism changes genetic information in the human transcriptome. A single-nucleotide mutation is often related to allele-specific human diseases. Those include albinism, cystic fibrosis, galactosemia, phenylketonuria, fragile X syndrome or Huntington's disease, and many more (Magner et al. 2016; Wu et al. 2020). Based on the results in Tables 1 and 2, the best thermodynamic differentiation could be expected for transversion-related SNPs when changing C to G. In that case, 7G RNA duplexes containing 7G-G are 2.24–2.64 kcal/mol more favorable than RNA duplexes containing inside 7G-C pair, and that corresponds to 60-70 fold increased binding of antisense oligonucleotide containing 7G to the allele with G compared to the allele with C.

## MATERIALS AND METHODS

### Chemical synthesis of protected N7-guanosine

Chemical synthesis of N7-guanosine in a 5–10 mmol scale was carried out using the method described in an earlier study (Boryski and Manikowski 1995). Condensation of N9,N2-diacetylguanine with tetraacetylribose was performed in anhydrous acetonitrile in the presence of tin (IV) chloride for 45–60 min at room temperature. After column chromatography purification, the yield of tertraacetyl-N7-guanosine was ∼50%. At room temperature, the *O*-acetyl group was deprotected with a mixture of methanol and aqueous ammonia. N2-Acetyl-N7-guanosine was treated with di-tert-butylsilyl bis(trifluoromethanesulfonate), resulting in simultaneous protection of 5′- and 3′-hydroxyls followed by protection of 2′-hydroxyl with tert-butyldimethylsilyl (tBDMSi) (Meynier et al. 2022). In the following synthesis step, the di-tert-butylsilyl protecting group was selectively removed (with pyridinium fluoride solution). After that, the dimethoxytrityl group (DMTr) was introduced at the 5′-position. That 5′-O-DMTr-2′-O-tBDMSi-N2-acetyl-N7-guanosine was converted into 3′-O-phosphoramidite using 2-cyanoethyl N,N,N′,N′-tetraisopropylphosphorodiamidite (McBride et al. 1986; Kierzek and Kierzek 2003). The structure of N7-guanosine was confirmed by various NMR methods and compared to guanosine (see Supplemental Materials and Methods; Supplemental Tables S4 and S5; Supplemental Figs. S8–S14).

### Oligonucleotide synthesis

Oligonucleotides were synthesized on a BioAutomation MerMade12 DNA/RNA synthesizer using β-cyanoethyl phosphoramidite chemistry and commercially available phosphoramidites (ChemGenes, GenePharma) (Beaucage and Caruthers 1981). A standard solid-phase RNA synthesis protocol was used to synthesize the oligonucleotides. For deprotection, oligoribonucleotides were treated with a mixture of 30% aqueous ammonia/ethanol (3/1 v/v) for 16 h at 55°C. Silyl protecting groups were cleaved by treatment with triethylamine trihydrofluoride. Deprotected oligonucleotides were purified by silica gel thin layer chromatography (TLC) in 1-propanol/aqueous ammonia/water (55/35/10 v/v/v) as described in detail previously (Xia et al. 1998; Kierzek and Kierzek 2003).

### UV melting experiments

The thermodynamic measurements were performed for nine various concentrations of 7G RNA duplex in the range 100–1 µM on JASCO V-650 UV/Vis spectrophotometer in buffer containing 1 M sodium chloride, 20 mM sodium cacodylate, and 0.5 mM Na_2_EDTA, pH 7 (Freier et al. 1986; Xia et al. 1998). Oligonucleotide single-strand concentrations were calculated from the absorbance above 80°C, and single-strand extinction coefficients were approximated by a nearest-neighbor model (Borer 1975; Richards 1975). Absorbance versus temperature melting curves were measured at 260 nm with a heating rate of 1°C/min from 0°C to 90°C on a JASCO V-650 spectrophotometer with a thermoprogrammer. The melting curves were analyzed, and the thermodynamic parameters were calculated from a two-state model with the program MeltWin 3.5 (McDowell and Turner 1996). For most duplexes, the Δ*H*° derived from $T_{M}^{-1}$ versus ln(*C*_*T*_/4) plots are within 15% of that derived from averaging the fits to individual melting curves, as expected if the two-state model is reasonable.

### NMR spectroscopy of RNA duplexes

All RNA duplexes were dissolved in a 10 mM sodium phosphate buffer (pH 6.8) containing 150 mM sodium chloride and 0.1 mM Na_2_EDTA for the NMR experiments. To ensure that RNA is present uniquely in the duplex form, the samples were further washed with the same buffer on an Amicon centrifugal filter with 3 kDa molecular weight cutoff, where any excess single-stranded RNA would pass through the pores of the Amicon membrane. NMR data was collected on a Bruker Avance III 700 MHz spectrometer equipped with a QCI CryoProbe. The resonance assignment of nonexchangeable aromatic and anomeric protons was achieved using standard procedures by analyzing NOESY spectra recorded in 100% D_2_O at 25°C and 35°C (Wijmenga and van Buuren 1998). The rest of the ribose protons were not assigned. The exchangeable protons were assigned using NOESY spectra measured in 90% H_2_O/10% D_2_O at 5°C and 15°C. For one of the duplexes bearing the 7G-G mismatch, an imino resonance belonging to the mismatched pair was observed. To assign it, this duplex was resynthesized with G14 residue ^15^N-enriched at the N1 position, and a ^15^N-selective 1D NMR spectrum was acquired. This spectrum (Supplemental Fig. S3) unambiguously identified the imino proton as belonging to G14. For the duplexes containing 7G-A, the ^1^H NMR spectrum and the ^1^H-^15^N selective optimized flip angle short transient-heteronuclear multiple quantum correlation (SOFAST-HMQC) NMR spectrum were acquired (Supplemental Figs. S1 and S2). All spectra were analyzed in NMRFAM-Sparky (Lee et al. 2015).

### Calculation of the free energies and enthalpies of the N7-methylguanine and N9-methylguanine tautomers

The details of the quantum mechanical (QM) free energy and enthalpy calculations using density functional theory (DFT) at B3LYP/6-311G(d) level of theory are provided in the supporting information (see Supplemental Materials and Methods; Supplemental Figs. S4 and S5). Three tautomers, that is, the 7G-N1H, 7G-N3H, and 7G-N1H,N2-imino tautomers (Fig. 4), were selected for the development of force field parameters and subsequent MD simulations based on the NMR observables and the energetic stability observed from DFT calculations.

### Force field parameters for N7G tautomers

The initial geometries of the 7G-N1H, 7G-N3H, and 7G-N1H,N2-imino tautomers were built using the molecular structure editor MOLDEN (Schaftenaar and Noordik 2000). The partial atomic charges were developed for the QM optimized geometries of 7G tautomers using a restrained electrostatic potential (RESP) fitting procedure (Bayly et al. 1993). The partial charges of the atoms of the residues except the C1′, H1′, and base atoms were kept the same as those for standard guanosine nucleoside during the partial charge calculation step. The glycosidic torsion parameters (χ_KOL0_) were developed using QM and molecular mechanical calculations for the 7G-N1H tautomer (Supplemental Figs. S15 and S16), and these parameters were used for the other two tautomers. The representative structures of the three 7G-tautomers in their nucleotide forms are shown in Supplemental Figure S17. The methods for developing partial atomic charges and reoptimization glycosidic torsion potential are described in detail in the Supplemental Materials and Methods. The force field parameters (AMBER ff99 + χ_KOL0_) are provided in the supporting information (Supplemental Appendix S1). These parameters (in combination with bsc0 γ parameters) were validated using 16 nsec REMD (replica exchange MDs) simulation of 7G-N1H nucleoside in 16 temperature replica windows (from 300°K to 400°K) following the same protocol as reported (Dutta et al. 2022). They were observed to reproduce the NMR data reported earlier (Supplemental Table S6; Baranowski et al. 2022).

### Preparation of the initial model structures for duplexes

All duplexes with unmodified residues were prepared using RNAComposer (Popenda et al. 2012; Sarzynska et al. 2023). To prepare duplexes with central 7G-A or 7G-G, the duplexes with central G-A, A-A, or G-G base pairs in *anti-anti* conformation were modified using UCSF Chimera (Pettersen et al. 2004). The AMBER RNA_OL3 parameters (Cornell et al. 1995; Pérez et al. 2007; Zgarbová et al. 2011) were used for regular RNA, and the FF99_χ_KOL0_ parameters (developed in this study, in combination with the bsc0 α/γ parameters) (Pérez et al. 2007) were used for 7G tautomers.

### Molecular dynamics simulations

The initial structures were solvated with transferable intermolecular potential three-point water model (TIP3P) water molecules in truncated octahedral boxes with a minimum distance of 11 Å between any atom of the solute and the edge of the periodic box, and the systems were then neutralized using Na^+^ ions. The energy minimization was performed using 200 steps of steepest descent followed by 300 steps of conjugate gradient. The energy minimized systems were then heated from 0°K to 300°K in 100 psec using constant volume Langevin dynamics with 1 psec^−1^ collision frequency and using 15 kcal/mol-Å^2^ position restraints for holding the duplexes. Then, the systems were equilibrated gradually, reducing the restraint on the solute in four steps (10, 5, 1, and 0.5 kcal/mol), each kept for 100 psec. Subsequently, the production run step was performed with interstrand Watson–Crick hydrogen bond distance restraints applied to the terminal base pairs to prevent end-fraying (where 0.1 Å movement from the equilibrium bond distance was allowed), according to Saenger (1984). During the equilibration steps and production runs, the Langevin thermostat with collision frequency 1.0 psec^−1^ was used to control the temperature, and the Berendsen barostat with default compressibility of the system and pressure relaxation time taup = 1.0 psec (ntb = 2). Simulations were carried out with a 2 fsec time step, an 8 Å nonbonded cutoff, and SHAKE constraints on bonds to hydrogen atoms. The long-range electrostatic interactions were considered using the particle mesh Ewald (PME) method. The production runs were carried out for 1 μsec for the simulations of the duplexes using the *pmemd.cuda* module of AMBER18 (Case et al. 2006).

### Analysis of MD trajectories

Root mean square deviations (RMSD) (from the centroid structure), distances, λ angles (C1′-C1′-N7 and C1′-C1′-N9), and occurrence of hydrogen bonds (with donor-acceptor distance ≤3 Ǻ and donor-hydrogen-acceptor angle ≥135°) were calculated using suitable utilities available within *cpptraj* using the last 800 nsec of the trajectories. Cluster analysis was performed using the average-linkage clustering algorithm within *cpptraj*. Water and ion occupancy maps were generated using the *grid* routine (with parameters *nx* = 100, *x*_spacing = 0.5, *ny* = 100, *y*_spacing = 0.5, *nz* = 100, *z*_spacing = 0.5) in *cpptraj*. For the estimation of the molecular mechanics force field-based base stacking and base-pairing energies using the *lie* utility, we considered a cutoff of 999.0 Å for both the electrostatic and van der Waals interactions. Here, the energies were calculated as the sum of pairwise electrostatic (ELEC) and van der Waals (VDW) interaction energies between the atoms of the bases. The duplex hybridization energies were estimated using the MM/3D-RISM approach, and only seven internal base pairs were considered during the calculations. The MM/3D-RISM energy calculations were performed using the single trajectory approach using the MM-PBSA.py script (in AmberTools18) (Case et al. 2006) using a similar bash script (Yildirim et al. 2023). Further details for the MM/3D-RISM calculations and the specific parameters used are provided in the Supplemental Materials and Methods section, along with an additional description of the method.

## SUPPLEMENTAL MATERIAL

Supplemental material is available for this article.
